# *Cryptosporidium* and *Giardia* in Africa: current and future challenges

**DOI:** 10.1186/s13071-017-2111-y

**Published:** 2017-04-20

**Authors:** Sylvia Afriyie Squire, Una Ryan

**Affiliations:** 1 School of Veterinary and Life Sciences, Murdoch University, Perth, Australia; 2 Council for Scientific and Industrial Research, Animal Research Institute, Accra, Ghana

**Keywords:** *Cryptosporidium*, *Giardia*, Africa, Molecular typing, HIV, Malnutrition, Climate change

## Abstract

*Cryptosporidium* and *Giardia* are important causes of diarrhoeal illness. Adequate knowledge of the molecular diversity and geographical distribution of these parasites and the environmental and climatic variables that influence their prevalence is important for effective control of infection in at-risk populations, yet relatively little is known about the epidemiology of these parasites in Africa. *Cryptosporidium* is associated with moderate to severe diarrhoea and increased mortality in African countries and both parasites negatively affect child growth and development. Malnutrition and HIV status are also important contributors to the prevalence of *Cryptosporidium* and *Giardia* in African countries. Molecular typing of both parasites in humans, domestic animals and wildlife to date indicates a complex picture of both anthroponotic, zoonotic and spill-back transmission cycles that requires further investigation. For *Cryptosporidium*, the only available drug (nitazoxanide) is ineffective in HIV and malnourished individuals and therefore more effective drugs are a high priority. Several classes of drugs with good efficacy exist for *Giardia*, but dosing regimens are suboptimal and emerging resistance threatens clinical utility. Climate change and population growth are also predicted to increase both malnutrition and the prevalence of these parasites in water sources. Dedicated and co-ordinated commitments from African governments involving “One Health” initiatives with multidisciplinary teams of veterinarians, medical workers, relevant government authorities, and public health specialists working together are essential to control and prevent the burden of disease caused by these parasites.

## Background

Infectious diarrhoea is a major cause of death in children under 5 years old in Africa [1]. Unsafe water supplies and inadequate levels of sanitation and hygiene increase the transmission of diarrhoeal diseases and despite ongoing efforts to enhance disease surveillance and response, many African countries face challenges in accurately identifying, diagnosing and reporting infectious diseases due to the remoteness of communities, lack of transport and communication infrastructures, and a shortage of skilled health care workers and laboratory facilities to ensure accurate diagnosis [2].

The enteric protozoan parasites *Cryptosporidium* and *Giardia* are important causes of diarrhoeal disease [3–6], with *Cryptosporidium* the most common diarrhoea-causing protozoan parasite worldwide [7]. The recent Global Enteric Multicenter Study (GEMS) and other studies to identify the aetiology and population-based burden of paediatric diarrhoeal disease in sub-Saharan Africa, revealed that *Cryptosporidium* is second only to rotavirus as a contributor to moderate-to-severe diarrhoeal disease during the first 5 years of life [8]. It has been estimated that 2.9 million *Cryptosporidium*-attributable cases occur annually in children aged < 24 months in sub-Saharan Africa [9] and infection is associated with a greater than two-fold increase in mortality in children aged 12 to 23 months [8].

*Giardia duodenalis* is the species infecting mammals, including humans, and is estimated to cause 2.8 × 10^8^ cases of intestinal diseases per annum globally [10, 11], with a higher prevalence in developing countries including Africa [5]. Most infections are self-limited but recurrences are common in endemic areas. Chronic infection can lead to weight loss and malabsorption [12] and is associated with stunting (low height for age), wasting (low weight for height) and cognitive impairment in children in developing countries [13–15]. Furthermore, acute giardiasis may disable patients for extended periods and can elicit protracted post-infectious syndromes, including irritable bowel syndrome and chronic fatigue [16].

In Africa, GEMS reported that *Giardia* was not significantly positively associated with moderate-to-severe diarrhoea [8]. However, experimental challenge studies unequivocally document that some strains of *G. duodenalis* can cause diarrhoea in healthy adult volunteers [17, 18], and a recent systematic review and meta-analysis of endemic paediatric giardiasis concluded that there is an apparently paradoxical association with protection from acute diarrhoea, yet an increased risk of persistent diarrhoea [19].

In addition to diarrhoea, both protozoans have been associated with abdominal distension, vomiting, fever and weight loss in mostly children and HIV/AIDS individuals [20–29]. Malnutrition, which impairs cellular immunity, is an important risk factor for cryptosporidiosis [30] and *Cryptosporidium* infection in children is associated with malnutrition, persistent growth retardation, impaired immune response and cognitive deficits [31–33]. The mechanism by which *Cryptosporidium* affects child growth seems to be associated with inflammatory damage to the small intestine [34]. Undernutrition (particularly in children) is both a sequela of, and a risk factor for, cryptosporidiosis [33, 35–40]. For both parasites, breast-feeding is associated with protection against clinical cryptosporidiosis and giardiasis [19, 41–43], even though it does not generally prevent acquisition of *Giardia* infection or chronic carriage [19].

Currently 31 *Cryptosporidium* species are considered valid [44–48]. Among these, more than 20 *Cryptosporidium* spp. and genotypes have been reported in humans, although *C. parvum* and *C. hominis* remain the most common [6, 48, 49]. *Giardia duodenalis* consists of eight assemblages (A to H) with different host specificities; Assemblage A in humans, livestock and other mammals; B in humans, primates and some other mammals, C and D in dogs and other canids; E mainly in hoofed animals including cattle, sheep and goats and more recently in humans; F in cats and humans; G in rats; and H in marine mammals [50, 51].

Infection may be acquired through direct contact with infected persons (person-to-person transmission) or animals (zoonotic transmission) and ingestion of contaminated food (foodborne transmission) and water (waterborne transmission) [6, 11, 52]. Numerous studies have demonstrated that respiratory cryptosporidiosis may occur commonly in both immunocompromised and immunocompetent individuals and that *Cryptosporidium* may also be transmitted *via* respiratory secretions [53]. Several studies also suggest that flies may play an important role in the mechanical transmission of *Cryptosporidium* and *Giardia* including human infectious species [54–64].

Relatively little is known about the epidemiology of cryptosporidiosis and giardiasis in African countries [65, 66], although a recent review of *Cryptosporidium* in Africa focussed on the epidemiology and transmission dynamics [66]. The purpose of this review is to compare the prevalence and molecular epidemiology of both *Cryptosporidium* and *Giardia* in Africa, with a focus on current and future challenges and to develop recommendations for better control of these important parasites.

## Diagnosis and prevalence of *Cryptosporidium* and *Giardia* in Africa

Morphological identification of *Giardia* and *Cryptosporidium* (oo)cysts in faecal samples by microscopy either directly or after the application of stains including Acid Fast, Lugol’s iodine and immunofluorescent antibody staining are the most widely used methods for diagnosis of these parasites in Africa due to their relatively low cost (Table 1). In-house and commercial immunoassays including copro-antigen tests kits, *Crypto-Giardia* immuno-chromatographic dipstick kits, faecal antigen ELISA kits ImmunoCard STAT and CoproStrip™ *Cryptosporidium* are also widely used either alone or in combination with other techniques for research purposes (Table 1). Studies on *Cryptosporidium* and *Giardia* have mostly been in children aged 0–16 years, at primary schools, with or without gastrointestinal symptoms or community-based studies, while others have involved different groups of individuals including both HIV/AIDS-positive and negative patients (Table 1). There are also studies on food handlers or vendors and high-risk individuals in close contact with animals such as national park staff, people living close to national parks, and farmers and their households as well as solid waste workers [67]. As a result of the different diagnostic methods utilised, the prevalence of *Cryptosporidium* and *Giardia* in different African studies varies widely (Table 1), with prevalence of < 1% in children and adults and > 72% in diarrhoeic patients reported for *Cryptosporidium* and < 1% in children and HIV-positive and negative patients and > 62% in primary school children (Table 1).

**Table 1:** Prevalence of *Cryptosporidium* and *Giardia* in African countries (2010–2016)

Country	Study population	Age group	Percentage positive for *Giardia* (No. positive/No. tested)	Percentage positive for *Cryptosporidium* (No. positive/No. tested)	Diagnostic technique	Genotyping (target gene)	Reference
Algeria	Patients with digestive disorders	≤ 80 years	3.6% (38/1042)	0.1% (1/1042)	Direct wet mount, formal ether concentration, iodine solution and acid fast staining	Not genotyped	[271]
Algeria	Sporadic cases	≤ 75 years	41.7% (25/542)	–	Iodine staining	Not genotyped	[272]
Angola	Children (with and without diarrhoea)	≤ 12 years	0.1% (44/328); 21.6% (73/338)	30.0% (101/337)	Iodine and acid fast staining, and immunoassay	Not genotyped	[273, 274]
Botswana	Children with diarrhoea	< 5 years		60.0% (45/75)	Not stated	Not genotyped	[41]
Burkina Faso	Patients with diarrhoea	All ages		26.5% (77/291)	Acid fast staining	Not genotyped	[275]
Cameroon	Individuals from Nloh and Bawa villages	Not specified	7.1% (14/197)	1.5% (3/194)	Immunoassay	Not genotyped	[276]
Cameroon	HIV/AIDS-positive and negative individuals	15–70 years	0.3% (1/396)	2.5% (10/396); 6.0% (12/200); 14.4% (46/320)	Direct microscopy, formal ether concentration, iodine and acid fast staining	Not genotyped	[277–279]
Central Africa Republic	Employees at Dzanga-Sangha Park	Not specified	2.1% (1/48)		PCR	*tpi*	[122]
Central African Republic	Children (with and without diarrhoea)	Not stated; < 5 years	15.6% (27/173); 4.4% (29/666)	10.4% (18/173); 7.7% (51/666)	Merthiolate iodine formaldehyde concentration, acid fast staining, immunoassay and multiplex PCR	Not genotyped	[29, 280]
Chad	Individuals from different regions	≤ 76 years	3.5% (16/462)		Merthiolate-iodine-formaldehyde concentration	Not genotyped	[281]
Chad	European military UN peace keepers	21–51 years	22.3% (55/247)		Direct microscopy and iodine staining	Not genotyped	[282]
Côte d’Ivoire	Individuals with intestinal disorders, persistent diarrhoea and controls	≥ 1 year	19.9% (61/307); 29.0% (39/136)	6.2% (19/307); 8.8% (12/136)	Direct microscopy, iodine staining, formol ethyl-acetate concentration, formalin-ether concentration, immunoassay and PCR	*bg*; 18S rRNA; COWP	[106, 283]
Côte d’Ivoire	Children	≤ 19 years	21.6% (66/306); 20.7% (25/121)		Direct microscopy, iodine staining, formol-ethyl acetate concentration, ether-concentration technique	Not genotyped	[284, 285]
Democratic Republic of Congo	HIV/AIDS-positive individuals	15–73 years	1.7% (3/175)	9.7% (17/175); 5.4% (13/242)	Modified Ritchie formalin-ether concentration, acid fast staining and PCR assay	18S rRNA	[28, 286]
Egypt	Individuals with livestock in their household	≤ 60 years		19.0% (55/290)	Acid fast staining	COWP	[108]
Egypt	Individuals with and without diarrhoea	All ages	12.5% (15/150) to 27.3% (75/330)	5.9% (23/391) to 72.2% (52/92	Direct microscopy, formalin-ethyl acetate sedimentation, acid fast, iodine and trichrome staining, immunoassay and PCR	COWP; *tpi*; *gdh*; *bg*	[105, 121, 131, 287, 288]
Egypt	Children (with and without diarrhoea)	< 18 years	18.9% (224/1187) to 29.2% (47/161)	2.1% (15/707) to 49.1% (81/165)	Direct microscopy, modified Ritchie’s biphasic concentration, iodine and acid fast staining, immunoassay and PCR	18S rRNA; TRAP-C2; COWP; *tpi*; *gdh*; *bg*	[26, 80, 95, 132, 167, 289–292]
Egypt	Mentally handicapped individuals	All ages	8.5% (17/200)	23.5% (47/200)	Trichrome and acid fast staining	Not genotyped	[293]
Egypt	Children with acute lymphoblastic leukemia and controls	≤ 15 years		18.2% (10/55)	Acid fast staining and serum immunoassay	Not genotyped	[294]
Egypt	Municipality solid-waste workers	21–59 years	3.8% (13/346)	23.4% (81/346)	Formol-ether concentration and acid fast staining	Not genotyped	[67]
Equatorial Guinea	HIV-positive and negative individuals	≤ 76 years	14.2% (44/310); 4.2% (14/333)	2.7% (9/333) to 18.1% (31/171)	Direct microscopy, formol-ether concentration, iodine and acid fast staining, Immunoassay and PCR	COWP	[152, 295, 296]
Ethiopia	HIV-positive and negative individuals	≤ 86 years	4.0% (15/378) to 7.9% (39/491)	8.5% (32/378) to 26.9% (140/520)	Direct microscopy, formol-ether concentration, iodine and acid fast staining and PCR	18S rRNA	[23, 267, 297–300]
Ethiopia	Children	≤ 15 years	4.6% (16/350) to 55.0% (216/393)	4.6% (18/393) to 7.3% (28/348)	Direct microscopy, formol-ether concentration, iodine and acid fast staining, immunoassay and PCR	18S rRNA; *tpi*; *gdh*; *bg*	[103, 125, 299, 301]
Ethiopia	Individuals with diarrhoea or gastrointestinal symptoms	≤ 80 years	10.9% (10/92)	1.1% (1/92); 7.6% (79/1034)	Direct microscopy, acid fast staining, immunoassay and PCR	18S rRNA; COWP; *gdh*; *bg*	[82, 99]
Ghana	Children (with and without diarrhoea/gastrointestinal symptoms)	≤ 17 years	1.0% (1/101) to 37.9% (455/1199)	4.9% (59/1199) to 8.5% (204/2400)	Direct microscopy, formol-ether concentration, iodine and acid fast staining, immunoassay and PCR	18S rRNA; *gdh*	[98, 135, 302–305]
Ghana	HIV-positive and negative individuals	All ages	12.6% (101/800)	8.2% (34/413); 9.0% (72/800)	Direct microscopy, formol-ether concentration, iodine and acid fast staining	Not genotyped	[306, 307]
Ghana	Food vendors	10–70 years	10.7% (15/140)		Direct microscopy, iodine staining and formalin-ether concentration	Not genotyped	[308]
Guinea-Bissau	Children from villages	≤ 7.5 years	56.0% (28/50)		Ritchie concentration method and PCR	18S rRNA; *bg*	[309]
Guinea Bissau	Children (with and without malnutrition)	< 5 years	33.9% (37/109)		Direct microscopy	Not genotyped	[310]
Kenya	Certified food-handlers	≥ 18 years	1.3% (4/312)		Iodine staining	Not genotyped	[311]
Kenya	HIV/AIDS-positive patients	≥ 18 years		34.1% (56/164)	Immunoassay and PCR	18S rRNA	[25]
Kenya	Individuals in villages	≤ 81 years	41.3% (329/796)	<1%	Multi-parallel qPCR	Not genotyped	[81]
Kenya	Children (with and without diarrhoea)	≤ 15 years	4.6% (98/2112) to 11.1% (109/981)	3.7% (36/981) to 9.8% (31/317)	Direct microscopy, formal-ether concentration, acid fast staining, immunoassay and PCR	18S rRNA; *tpi*; *gdh*; *bg*	[71, 88, 115, 136, 312, 313]
Libya	Children with diarrhoea	≤ 5 years	1.3% (3/239)	2.1% (5/239)	Immunoassays	Not genotyped	[314]
Libya	Individuals with diarrhoea	Not specified	1.3% (4/305)	2.3% (7/305)	Direct microscopy, iodine, eosin and acid fast staining, concentration methods	Not genotyped	[315]
Madagascar	Children (with and without diarrhoea)	< 5 years	11.7% (314/2692)		Direct microscopy and iodine staining	Not genotyped	[316]
Madagascar	Individuals from rural southeastern Madagascar	Not specified		0.8% (1/120)	PCR-RFLP	18S rRNA	[97]
Mali	European soldiers	Not specified	3.8% (2/53)	5.7% (3/53)	Multiplex real time PCR	Not genotyped	[317]
Morocco	Children (urban and rural)	5–15 years	12.5% (84/673)		Iodine, acid fast and Giemsa staining and Faust’s and Ritchie’s concentration methods	18S rRNA; *gdh*	[318]
Mozambique	HIV-positive and negative individuals	< 5 years	6.5% (6/93)		Direct microscopy and Ritchie’s concentration method	Not genotyped	[319]
Nigeria	Children (with and without diarrhoea or gastroenteritis)	≤ 15 years	37.2% (74/199)	1.0% (2/199) to 46.8% (88/188)	Direct microscopy, acid fast staining, immunoassay and qPCR (18S rRNA gene)	18S rRNA	[86, 87, 141, 320–323]
Nigeria	HIV/AIDS-positive and negative individuals	≤ 80 years	1.9% (9/476) to 3.2% (5/157)	1.9% (3/157) to 35.9% (171/476)	Direct microscopy, acid fast staining, PCR-RFLP	18S rRNA; *tpi*; ITS rDNA	[83, 101, 166, 324, 325]
Rwanda	Children	≤ 18 years	35.7% (222/622); 59.7% (366/583)		Direct microscopy, ether-based concentration, real-time PCR assays and multiplex qPCR	*tpi*	[22, 24, 326]
São Tomé and Príncipe	Children (with and without diarrhoea)	≤ 10 years	8.3% (29/348); 41.7% (185/444)	5.5% (19/348)	Direct microscopy, modified water-ether sedimentation, formol-ether concentration, iodine and acid fast staining and PCR-RFLP	18S rRNA; *tpi*	[96, 327]
Senegal	Children (with and without diarrhoea)	< 15 years		6.13% (23/375)	Acid fast stain and immunoassay	Not genotyped	[328]
South Africa	Children (with and without diarrhoea)	≤ 11 years	9.9% (16/162)	5.6% (8/143); 12.2% (54/442)	Acid fast staining and PCR	18S rRNA	[89, 154, 329]
South Africa	HIV-positive individuals	All ages	11.9% (18/151)	65.5% (165/252); 26.5% (40/151)	Acid fast stain, immunoassay, qPCR, PCR (18S rDNA) and loop-mediated isothermal amplification (LAMP)	Not genotyped	[330, 331]
Sudan	Inhabitants of rural areas	All ages		13.3% (115/866)	Acid fast staining	Not genotyped	[332]
Sudan	Children (with and without diarrhoea)	≤ 13 years and primary school-aged	10.1% (91/900) to 33.4% (167/500)		Direct microscopy and concentration technique	Not genotyped	[333–336]
Sudan	Food handlers	4–57 years	20.5% (16/259)		Direct microscopy	Not genotyped	[337]
Sudan	Individuals with diarrhoea	1–80 years	22.0% (22/100)		Direct microscopy, formal ether concentration technique	Not genotyped	[338]
Tanzania	Individuals with and without diarrhoea	All ages	6.9% (218/3152); 53.4% (93/174)	1.1% (2/174)	Formol-ether-concentration technique, iodine and acid fast staining, qPCR and conventional PCR	18S rRNA	[339, 340]
Tanzania	Children (with and without diarrhoea)	< 5 years, school going age	1.9% (5/270) to 62.2% (28/45)	10.4% (131/1259); 18.9% (51/270)	Multiplex real-time PCR and immunoassays	18S rRNA; *gdh*	[72, 123, 341]
Tanzania	Individuals living in and around Gombe National Park	Not stated		4.3% (8/185)	PCR-RFLP	18S rRNA	[102]
Tanzania	HIV-positive individuals	30–43 years	9.6% (8/83)		Direct microscopy and iodine staining	Not genotyped	[342]
Tunisia	Children (with and without diarrhoea)	< 5 years		1.7% (7/403)	Acid fast staining	18S rRNA	[114]
Uganda	Individuals from villages near Kibale National	≤ 75 years	40.7% (44/108)	32.4% (35/108)	Real-time PCR, nested PCR	COWP; *ef1-α*; *gdh*; 18S rRNA; *tpi*	[107, 120]
Uganda	HIV-positive patients with Diarrhoea	10–69 years		3.6% (4/111)	Acid fast staining	Not genotyped	[343]
Uganda	Children (non-symptomatic and HIV seronegative)	≤ 12 years	20.1% (86/427)	12.5% (116/926)	Direct microscopy, acid fast staining and PCR	18S rRNA; *bg*; *gdh*; *tpi*	[344, 345]
Zambia	Pre-school children	≤ 7 years	29.0% (117/403) to 29.0% (228/786)	28.0% (113/403) to 30.7% (241/786)	Immunofluorescence stain	Not genotyped	[128, 346, 347]
Zimbabwe	Individuals in urban areas	All ages	2.7% (8/300)	6.3% (19/300)	Acid fast staining	Not genotyped	[348]

*Abbreviations*: *bg*, beta-giardin; COWP, *Cryptosporidium* oocyst wall protein gene; *ef1*-*α*, elongation factor 1-alpha; *gdh*, glutamate dehydrogenase; ITS, internal transcribed spacer; 18S rRNA, 18S ribosomal ribonucleic acid; *tpi*, triose phosphate isomerase gene; TRAP, thrombospondin-related adhesive protein

The immune status of the host, both innate and adaptive immunity, has a major impact on the severity of cryptosporidiosis and giardiasis and their prognosis [3, 50, 51, 68]. With both parasites, immunocompetent individuals typically experience self-limiting diarrhoea and transient gastroenteritis lasting up to 2 weeks and recover without treatment, suggesting an efficient host anti-*Cryptosporidium/Giardia* immune responses [3, 68]. With cryptosporidiosis, immunocompromised individuals including HIV/AIDS patients (not treated with antiretroviral therapy) often suffer from intractable diarrhoea, which can be fatal [69]. HIV status is an important host risk factor particularly for cryptosporidiosis and although *Cryptosporidium* is an important pathogen regardless of HIV-prevalence [8], HIV-positive children are between three and eighteen times more likely to have *Cryptosporidium* than those who are HIV-negative [70–72]. The unfolding HIV/AIDS epidemic in African countries, with > 25 million adults and children infected with HIV/AIDS in 2015 [2], is a major contributor to the increased prevalence of cryptosporidiosis and giardiasis in Africa.

The impact of malnutrition usually falls mainly on children under 5 years of age [73] and malnutrition is an important risk factor for both diarrhoea and prolonged diarrhoea caused by *Cryptosporidium* and *Giardia* [19], with significantly higher rates of *Cryptosporidium* infection in malnourished children controlling for HIV status [33, 39, 74, 75].

## Molecular detection and characterisation of *Cryptosporidium* and *Giardia*

Molecular tools for the detection and characterisation of these parasites are increasingly being used however, particularly for research purposes due to increased specificity and sensitivity and the ability to identify species [29, 76–81]. The most commonly used genotyping tools for *Cryptosporidium* in Africa are PCR and restriction fragment length polymorphism (RFLP) and/or sequence analysis of the 18S rRNA gene [23, 25, 28, 72, 82–103] (Table 1), although some studies have relied on the *Cryptosporidium* oocyst wall protein (COWP) gene [26, 82, 92, 100, 104–108], which is not as reliable as the 18S locus at identifying and differentiating *Cryptosporidium* species [109]. Subtyping of *Cryptosporidium* has been conducted mainly at the glycoprotein 60 (*gp60*) gene locus [23, 26, 82, 93–95, 98, 100, 102, 108, 110–115] (Table 2) while others targeted the heat shock protein 70 (HSP70) gene [91, 92, 100, 116–118]. Genotyping of *Giardia* in Africa, has mainly been conducted using the triose-phosphate isomerase (*tpi*) gene, beta-giardin (*bg*) and glutamate dehydrogenase (*gdh)* genes, either alone or using a combination of two or three loci [80, 99, 103, 119–124] (see Tables 1, 3 and 4).

**Table 2:** *Cryptosporidium* species and subtypes reported in humans from Africa

Country	Patient group	*Cryptosporidium* species (Prevalence in %)^a^	*gp60* subtypes	Reference
Botswana	Children with diarrhoea (< 5 years)	*C. parvum* (50.0)		[41]
		*C. hominis* (41.0)		
Côte d’Ivoire	Individuals with intestinal disorders	*C. meleagridis* (75.0)		[106]
		*C. parvum* (16.7)		
		*C. hominis* (8.3)		
Democratic Republic of Congo	HIV-infected adults	*C. hominis* (80.0)		[28]
		*C. parvum* (20.0)		
Egypt	Individuals with livestock in their household	*C. hominis* (75.0)		[108]
		*C. parvum* (25.0)	IIdA20G1	
Egypt	Children with diarrhoea (≤ 10 years)	*C. hominis* (60.5)		[26]
		*C. parvum* (38.3)		
Egypt	Diarrhoeal patients	*C. hominis* (97.0)		[131]
		*C. parvum* (3.0)		
Egypt	Children with diarrhoea (1–14 years)	*C. parvum* (82.0)		[290]
		*C. hominis* (12.0)		
		Mixed infections (6.0)		
Egypt	Children with diarrhoea (< 10 years)	*C. hominis* (60.5)		[95]
		*C. parvum* (38.3)	IIaA15G1R1; IIaA15G2R1; IIdA20G1^a^	
		*C. parvum* + *C. bovis* (1.2)		
Egypt	Diarrhoeal patients	*C. hominis* (60.0)		[105]
		*C. parvum* (20.0)		
		*C. hominis* + *C. parvum* (20.0)		
Egypt	Gastrointestinal symptomatic patients	*C. parvum* (66.7)		[156]
		*C. hominis* (27.7)		
		*C. meleagridis* (5.6)		
Egypt	Immunocompromised patients	*C. parvum* (68.4)		[349]
		*C. hominis* (26.3)		
		*C. parvum* + *C. hominis* (5.3)		
Equatorial Guinea	HIV-infected and immunocompetent patients	*C. parvum* (52.9)		[150]
		*C. hominis* (44.1)		
		*C. meleagridis* (2.9)		
Equatorial Guinea	HIV-seropositive patients	*C. parvum* (54.8)	IIcA5G3^b^; IIeA10G1	[152]
		*C. hominis* (50.0)	IaA18R3^b^; IaA24R3; IbA13G3; IdA15; IdA18^b^	
		*C. meleagridis* (3.2)		
Ethiopia	Diarrhoeal patients (14–71 years)	*C. parvum* (95.1)	IIaA15G2R1^b^; IIaA16G2R1; IIaA16G1R1	[82]
		*C. hominis* (2.4)	IbA9G3	
		*C. hominis* + *C. parvum* (2.4)		
Ethiopia	HIV/AIDS patients	*C. parvum* (65.7)	IIaA13G2R1; IIaA14G2R1; IIaA15G2R1^b^; IIaA16G2R1; IIaA16G3R1; IIaA17G2R1; IIaA18G2R1; IIaA19G1R; IIbA12; IIcA5G3a; IIdA17G1; IIdA19G1; IIdA22G1; IIdA24G1; IIeA12G1; If-like	[23]
		*C. hominis* (17.9)	IbA10G2; IdA20^b^; IdA24; IdA26; IeA11G3T3	
		*C. viatorum* (7.1)		
		*C. felis* (3.6)		
		*C. meleagridis* (2.1)		
		*C. canis* (1.4)		
		*C. xiaoi* (1.4)		
		*C. hominis* + *C. parvum* (0.7)		
Ethiopia	Patients	*C. hominis* (100)	IbA9G3	[99]
Ethiopia	*Cryptosporidium*-positive (7 months to 27 years)	*C. viatorum* (90.0)	XVaA3d	[161]
Ethiopia	Children (6–15 years)	*C. hominis* (40.0)		[103]
		*C. viatorum* (40.0)		
		*C. parvum* (20.0)		
Ghana	Children (≤ 5 years)	*C. parvum* (100)		[142]
Ghana	Children with and without gastrointestinal symptoms	*C. hominis* (58.0)	IaA15T1R3; IaA15G1R4; IaA17; IaA18R3; IaA19R3; IaA21R3; IaA22R3; IaA24R3; IbA13G3^b^; IdA15; IeA11G3T3	[98]
		*C. parvum* (42.1)	IIcA5G3a^b^; IIcA5G3b; IIeA10G1; IIeA10G2	
Kenya	HIV patients	*C. hominis* (66.7)		[146]
		*C. parvum* (16.7)		
		*C. meleagridis* (16.7)		
Kenya	Not stated	*C. hominis* (*C. parvum* human*)* (90.0)	Ib; Ie^b^	[155]
		*C. meleagridis* (10.0)		
Kenya	An HIV-infected adult with diarrhoea	*C. muris* “rock hyrax”		[139]
Kenya	HIV-positive and negative children and adults	*C. parvum* human (69.7)		[147]
		*C. parvum* bovine (21.2)		
		*C. meleagridis* (3.0)		
		*C. muris* (3.0)		
Kenya	Children (< 5 years)	*C. hominis* (87.4)		[129]
		*C. parvum* (8.6)		
		*C. canis* (1.7)		
		*C. felis* (1.1)		
		*C. muris* (0.6)		
		*C. meleagridis* (0.6)		
Kenya	Children (≤ 5 years)	*C. hominis* (82.1)		[88]
		*C. parvum* (17.9)		
Kenya	HIV/AIDS patients with and without diarrhoea (≥ 18 years)	*C. hominis* (60.7)	Ia; Ib^b^; Id; Ie; If	[25]
		*C. parvum* (23.2)	IIa^b^; IIb; IIe^b^; IIc	
		*C. canis* (7.1)		
		*C. meleagridis* (5.3)		
		*C. suis* (3.6)		
Kenya	HIV infected and uninfected children (5 years)	*C. hominis* (82.8)	IaA25R5; IaA27R3; IaA30R3; IaA7R1; IbA9G3; IbA9G3R2; IdA22; IdA24; IdA19; IdA25; IdA21; IdA20; IdA17G1; IdA18; IdA15G1; IdA23G1; IeA11G3T3R1^b^; IeA11G3T3; IfA19G1; IfA14G1; IfA12G1	[115]
		*C. parvum* (11.9)	IIcA5G3R2	
		*C. felis* (2.6)		
		*C. meleagridis* (1.3)		
		*C. parvum* + *C. hominis* (0.7)		
Kenya	*Cryptosporidium* spp.-positive female (25 years)	*C. viatorum*	XVaA3d	[161]
Kenya	Children (≤ 5 years)	*C. hominis* (38.9)		[153]
		*C. parvum* (36.1)		
		*C. meleagridis* (16.7)		
		*C. canis* (5.6)		
		Unknown genotype (2.8)		
Madagascar	Children with acute diarrhoeal disease (≤ 16 years)	*C. hominis* (91.7)	IaA22R3; IdA15G1^b^; IeA11G3T3	[350]
		*C. parvum* (8.3)	IIcA5G3	
Madagascar	Humans from rural south-eastern Madagascar	*C. suis* (100)		[97]
Malawi	HIV-positive and negative children	*C. hominis* (63.6)		[147]
		*C. parvum* (36.4)		
Malawi	Children HIV-positive and seronegative (≤ 30 months)	*C. hominis* (95.3)	Ia; Ib; Id^b^; Ie	[351]
		*C. parvum* (4.7)	IIc; IIe	
Malawi	Diarrhoeal children (< 5 years)	*C. hominis* (58.1)		[149]
		*C. parvum* (23.3)		
		*C. parvum* + *C. hominis* (2.3)		
		*C. parvum*/*C. hominis* (9.3)		
		*C. meleagridis* (4.7)		
		*C. muris*/*C. andersoni* (2.3)		
Nigeria	HIV-infected (majority) and non-infected patients	*C. hominis* (47.2)	IaA14R3; IaA16R3; IaA24R3; IaA25R3; IbA13G3; IeA11T3G3^b^	[83]
		*C. parvum* (44.4)	IIcA5G3a; IIcA5G3h; New subtype family 1^b^; New subtype family 2	
		*C. felis* (5.6)		
		*C. canis* (2.8)		
Nigeria	Children (6 months to 6 years)	*C. hominis* (44.2)	IaA18R2; IaA22R2; IaA24R2; IaA25R2; IaA28R2; IaA21R1; IbA10G2; IbA13G3^b^; IdA11; IdA17; IeA11G3T3^b^; Ih (Novel subtype); IhA14G1	[141]
		*C. parvum* (32.5)	IIaA15G2R1; IIaA16G1R1; IIcA5G3a^b^; IIcA5G3b; IIiA11; IImA14G1	
		*C. parvum* plus *C. hominis* (5.2)		
		*C. meleagridis* (6.5)		
		*C. cuniculus* (*C.* rabbit genotype) (6.5)		
		*C. ubiquitum* (*C.* cervine genotype) (3.9)		
		*C. canis* (1.3)		
Nigeria	Children (19.5–72 months)	*C. hominis* (37.3)		[86]
		*C. parvum* (35.3)		
		*C. parvum* + *C. hominis* (7.8)		
		*C. meleagridis* (7.8)		
		*C. cuniculus* (*C.* rabbit genotype) (3.9)		
		*C. ubiquitum* (*C.* cervine genotype) (2.0)		
		*C. canis* (2.0)		
Nigeria	Diarrhoeal and non-diarrhoeal children (1 to > 12 months)	*C. hominis* (60.0)	IaA24R3^b^; IbA13G3	[87]
		*C. parvum* (40.0)	IIcA5G3k	
Nigeria	Patients (2 months to 70 years)	*C. hominis* (66.7)	IaA23R3; IaA25R3	[166]
		*C. parvum* (33.3)	IIeA10G1	
Nigeria	HIV-infected patients (22–65 years)	*C. hominis* (50.0)	IeA11G3T3	[101]
		*C. parvum* (25.0)	IIcA5G3k	
		*C. meleagridis + C. hominis* (25.0)		
São Tomé and Príncipe	Children (2 months to 10 years)	*C. hominis* (73.7)	IeA11G3T3R1; IeA11G3T3; IaA27R3^b^; IaA23R3	[96]
		*C. parvum* (26.3)	IIdA21G1a; IIdA26G1^b^; IIaA16G2R1; IIaA15G2R1	
South Africa	Children (< 5 years)	*C. hominis* (75.0)	IbA12G3R2; IbA10G2; IeA11G3T3	[154]
		*C. meleagridis* (25.0)		
South Africa	Children (< 5 years)	*C. hominis* (76.0)	IaA20R3; IaA25G1R3; IaA17R3; IbA9G3; IbA10G1; IdA20; IdA25IdA26; IdA24; IeA11G3T3^b^; IfA14G1; IfA12G1	[89]
		*C. parvum* (20.0)	IIbA11; IIcA5G3b^b^; IIeA12G1	
		*C. meleagridis* (4.0)	IIIdA4	
South Africa	School children and hospital patients (≤ 88 years)	*C. hominis* (81.8)		[77]
		*C. parvum* (18.2)		
South Africa	HIV-infected children	*C. hominis* (75.0)	Ia; Ib; Id; Ie	[352]
		*C. parvum* (25.0)	IIc	
Tanzania	People living in and around Gombe National Park	*C. hominis* (100)	IfA12G2	[102]
Tanzania	Diarrhoeal children and controls (< 2 years old)	*C. hominis* (84.7)		[72]
		*C. parvum* (7.6)		
		*C. parvum* or *C. hominis* (7.6)		
Tanzania	HIV patients (18–65 years)	*C. hominis* (71.4)		[162]
		*C. parvum* (28.6)		
Tunisia	Children (< 5 years old)	*C. parvum* (57.1)	IIaA15G2R1^b^; IIdA16G1	[114]
		*C. meleagridis* (42.9)		
Tunisia	Children with primary immune deficiencies	*C. hominis* (40.0)		[151]
		*C. parvum* (20.0)		
		*C. meleagridis* (20.0)		
		*C. hominis*/*C. meleagridis* (20.0)		
Tunisia	Immunocompetent and immunodeficiency children	*C. parvum* (42.1)		[140]
		*C. hominis* (36.8)		
		*C. meleagridis* (21.1)		
Uganda	Volunteers (1.9 months to 75 years)	*C. parvum* (97.1)		[107]
		*C. parvum* or *C. hominis* or *C. cuniculus* (2.9)		
Uganda	Children (2–84 months)	*C. hominis* (74.4)	Ia; Ib; Id; Ie^b^	[148]
		*C. parvum* (18.3)	IIc^b^; IIg; IIh; Iii	
		*C. hominis* + *C. parvum* (3.7)		
		C. *meleagridis* (3.7)		
Uganda	Children with persistent diarrhoea, with and without HIV/AIDS (< 6 months)	*C. hominis* (73.7)		[70]
		*C. parvum* (18.4)		
		*C. parvum* + *C. hominis* (3.9)		
		*C. meleagridis* (3.9)		
Uganda	People who share habitats with free-ranging gorillas	*C. parvum* (100)		[191]
Zambia	Dairy farm workers and their household members	*C. parvum* (80.0)		[118]
		*C. hominis* (20.0)		

^a^Prevalence (percentage of species identified out of the total number of species genotyped per animal)

^b^Dominant *gp60* subtype

**Table 3:** *Giardia duodenalis* assemblages and subtypes reported in humans from Africa

Country	Patient group	Assemblage (Prevalence in %)^a^	Subtype	Reference
Algeria	Children (8 and 13 years)	A (37.5)	AI; AII^b^; Novel subtype	[119]
		B (56.3)	BIII; BIV; Novel subtypes	
		A + B (6.3)		
Central African Republic	Dzanga-Sangha Protected Areas Park employees	A (100)	AII	[122]
Côte d’Ivoire	Patients with intestinal disorders (2–63 years)	A (19.6)		[106]
		B (76.8)		
		A + B (3.6)		
Côte d’Ivoire	Patients with intestinal disorders (2–63 years)	A (34.4)		[106]
		B (59.0)		
		A + B (6.6)		
Egypt	Diarrhoeal patients (2–70 years)	A (6.7)	AI/AII	[121]
		B (80.0)	BIII; B/BIII; BIV; BIII/BIV^b^	
		A + B (6.7)		
		C (6.7)		
Egypt	Diarrhoeal patients (All ages)	A (18.8)		[132]
		B (81.2)		
Egypt	Children (< 10 years)	A (30.4)	AII	[80]
		B (52.2)		
		E (8.7)		
		A/B (4.3)		
		A/E (4.3)		
Egypt	Children (5–12 years)	A (77.1)	AII	[167]
		B (22.9)	BIII	
Egypt	Children with and without diarrhoea (1.5–12 years)	E (100)		[169]
Egypt	Diarrhoeal patients (4–65 years)	A (75.6)	AI^b^; AII	[165]
		B (19.5)		
		A + B (5.0)		
Egypt	Humans	A (5.0)		[168]
		B (80.0)		
		E (15.0)		
Ethiopia	Children (6–15 years)	A (17.9)	AII^b^; AII/AIII	[103]
		B (82.1)	BIII; BIV^b^; BIII/BIV	
Ethiopia	Children (≤ 14 years)	A (22.9)	AII^b^	[301]
		B (77.1)	BIV; Novel subtypes^b^	
Ethiopia	Hospital patients (0.5–80 years)	B (100)	BIII; BIV^b^	[99]
Ethiopia	Human isolates from urban and rural areas	A (52.5)		[164]
		B (22.0)		
		A + F (11.9)		
		A + B (13.6)		
Ghana	Children (≤ 5 years)	B (100)	BIII	[305]
Guinea-Bissau	Children (8.3 months to 7.5 years)	A (11.5)	AII	[309]
		B (84.6)		
Kenya	Diarrhoeal children (≤ 5 years)	A (1.4)	AII	[313]
		B (88.9)	BIII; BIV; BIII/BIV^b^	
		A + B (9.7)		
Morocco	Urban and rural school children (5–15 years)	A (18.1)	AII	[318]
		B (81.8)	BIII; BIV^b^	
Nigeria	Hospital patients (2 months to 70 years)	A (100)	AII	[166]
Rwanda	Largely asymptomatic children (< 5 years)	A (13.5)	AI; AII^b^	[22]
		B (85.9)		
		A + B (0.5)		
Rwanda	Children (< 5 years)	A (14.0)		[24]
		B (86.0)		
São Tomé and Príncipe	Children (2 months to 10 years)	A (55.5)		[96]
		B (45.5)		
Tanzania	Diarrhoeal and non-diarrhoeal outpatients (≤ 71 years)	A (6.7)		[340]
		B (88.8)		
		A + B (4.5)		
Tanzania	Diarrhoeal children and controls (< 2 years)	A (50.0)		[72]
		B (50.0)		
Tanzania	Primary school children	A (21.4)	AII	[123]
		B (78.6)	BIII; BIV^b^	
Uganda	Individuals living around Kibale National Park	A	AII	[120]
		B	BIII; BIV	
Uganda	Apparently healthy children (0–12 years)	A (14.7)	AII	[345]
		B (73.5)		
		A + B (11.8)		
Uganda	Individuals sharing gorilla habitats	A (100)		[163]

^a^Prevalence (percentage of species identified out of the total number of species genotyped per animal)

^b^Dominant subtype

**Table 4:** Prevalence and *Giardia duodenalis* assemblages and subtypes reported in animals from Africa

Country	Animal type	Patient group	Prevalence^a^ (No. positive/No. tested)	*Giardia duodenalis* assemblage (Prevalence in %)^b^	Subassemblage	Reference
Central African Republic	Wildlife	Wild western lowland gorillas (*Gorilla gorilla gorilla*)	2.0% (4/210)	A (100)	AII	[122]
	Domestic	Goat (*Capra aegagrus hircus*)	11.1% (1/9)	E (100)		
Côte d’Ivoire	Domestic	Dog (*Canis familiaris*)	54.5% (6/11)	A (33.3)		[106]
				A + B (16.7)		
				C (33.3)		
				D (33.3)		
		Goat (*Capra aegagrus hircus*)	50% (1/2)	A + B (100)		
		Duck (*Cairina moschata*)	33.3% (1/3)	A + B (100)		
		Chicken (*Gallus gallus*)	58.1% (18/31)	A (38.9)		
				B (38.9)		
				A + B (22.2)		
Egypt	Domestic	Cattle (*Bos taurus*)	6.7% (40/593)	A (15.4)	AI; AII	[80]
				E (82.7)		
				A/E (1.9)		
		Buffalo (*Bubalus bubalis*)	4.7% (10/211)	A (20.0)		
		Both cattle and buffalo	53.2% (424/804) by RT-PCR	E (80.0)		
Egypt	Domestic	Cattle (*Bos taurus*)	8.7% (4/46)	E (100)		[169]
Egypt	Domestic	Calves (*Bos taurus*)	30.8% (25/58)	A (20.0)		[175]
				E (80.0)		
Egypt	Wild and cultured	Fish (*Tilapia nilotica*; *Mugil cephalus*)	3.3% (3/92)	A (100)		[171]
Ghana	Wildlife	Colobus monkey (*Colobus vellerosus*)	69.2% (74/107)	B (100)		[189]
Rwanda	Wildlife	Mountain gorillas (*Gorilla beringei beringei*)	8.5% (11/130)	B (100)	BIV^c^; BIII	[124]
	Domestic	Cattle (*Bos taurus*)	5.9% (8/135)	E (100)		
Tanzania	Domestic	Zebus cattle (*Bos primigenius indicus*)	21.1% (4/19)	A (75.0)	BIV	[123]
				B (25.0)		
		Goats (*Capra aegagrus hircus*)	22.0% (9/41)	E (66.7)	BIV	
				B (22.2)		
				A (11.1)		
Uganda	Wildlife	Gorilla (*Gorilla gorilla beringei*)	2.0% (2/100)	A (100)		[163]
	Domestic	Cattle (*Bos taurus*)	10.0% (5/50)	A (100.0)		
Uganda	Wildlife	Red colobus (*Procolobus badius tephrosceles*)	11.1%	AII		[120]
				BIV		
				E		
	Domestic	Livestock (cattle, *Bos taurus* and *Bos indicus*, *n* = 25; goats, *Capra aegagrus hircus*, *n* = 57; and sheep, *Ovis aries*, *n* = 7)	12.4%	E		

^a^Prevalence (percentage of number positive out of the total number of animal species tested)

^b^Prevalence (percentage of species identified out of the total number of species genotyped per animal)

^c^Dominant subassemblage

Risk factor analysis have associated a higher prevalence of *Cryptosporidium* and/or *Giardia* with various factors including contact with animals and manure [25, 26, 125], the location of an individual such as living in villages *versus* cities, drinking underground or tap water [25, 26, 80], living in a household with another *Cryptosporidium*-positive person [102], and eating unwashed/raw fruit [27].

Precipitation is thought to be a strong seasonal driver for cryptosporidiosis in tropical countries [126, 127]. Many studies in Africa have reported a higher prevalence of *Cryptosporidium* during high rainfall seasons. For example, studies in Ghana (West Africa), Guinea-Bissau (West Africa), Tanzania (East Africa), Kenya (East Africa) and Zambia (southern Africa) have reported a higher prevalence of *Cryptosporidium* just before, or at the onset of the rainy season and a higher prevalence of *Giardia* in cool seasons in Tanzania [20, 72, 128]. However, other studies from Rwanda, Malawi, Kenya and South Africa have reported a higher prevalence of cryptosporidiosis at the end of rainy seasons and beginning of the drier months [129, 130]. Studies in Egypt (North Africa) reported a peak prevalence for both *Cryptosporidium* and *Giardia* during summer (drier months) with a second peak in winter for *Giardia* [131, 132]. It is possible that the apparent seasonality of human disease, is reflective of different transmission pathways, hosts, and/or *Cryptosporidium* and *Giardia* species in different locations. As climate change occurs, transmission patterns of many waterborne diseases may shift, and studies in African locations with unusual seasonality patterns will help inform our understanding of what climate change may bring.

*Cryptosporidium* and *Giardia* co-infections and co-infections with other pathogens have been observed in numerous studies in Africa [21, 129, 133–136]. In Kenya, polyparasitism was more common in patients with diarrhoea than those with single infections of intestinal parasites [137]. Multiple infections could impact on the host’s response to infection, as synergistic interaction between co-infecting pathogens has been shown to enhance diarrhoea pathogenesis. For example, in Ecuador, South America, simultaneous infection with rotavirus and *Giardia* resulted in a greater risk of having diarrhoea than would be expected if the co-infecting organisms acted independently of one another [138].

## *Cryptosporidium* and *Giardia* species reported in humans in Africa

Genotyping of *Cryptosporidium* species in Africa have identified at least 13 species and genotypes in humans including *C. hominis*, *C. parvum*, *C. meleagridis, C. ubiquitum*, *C. viatorum*, *C. andersoni*, *C. bovis*, *C. canis*, *C. cuniculus*, *C. felis*, *C. muris*, *C. suis* and *C. xiaoi* [23, 25, 26, 86, 97, 101, 102, 115, 139–142] (see Table 2).

*Cryptosporidium hominis* and *C. parvum* are the main species infecting humans [6, 143–145]. Out of the 56 molecular studies in African countries analysed, *C. hominis* was the most prevalent (2.4–100%) *Cryptosporidium* species in humans in 38 of the studies followed by *C. parvum* (3.0–100%) in 13 studies and *C. meleagridis* (75%) in one study, *C. viatorum* and C. *hominis* (40% each) in one study and a single species of *C. muris*, *C. suis* and *C. viatorum* in the remaining three studies (See Table 2).

*Cryptosporidium meleagridis* is also recognized as an important human pathogen in many African countries including Kenya, Cote d’Ivoire, Equatorial Guinea, Ethiopia, Malawi, Nigeria, South Africa, Tunisia and Uganda [23, 25, 70, 86, 89, 101, 106, 114, 129, 140, 141, 146–154]. In immunocompromised individuals, the prevalence of *C. meleagridis* can reach 75% (3/4 of samples typed) [23, 25, 101, 146, 147, 151, 152], but also 75% (9/12 of samples typed) in immunocompetent individuals [86, 106, 114, 141, 149, 153, 155, 156]. In comparison, the prevalence of *C. meleagridis* in the developed world is ~1% [66].

Other *Cryptosporidium* species including *C. viatorum*, *C. canis*, *C. muris*, *C. felis*, *C. suis* and *C. xiaoi* have been detected in immunocompromised individuals [23, 83, 139, 147] and *C. andersoni*, *C. bovis*, *C. viatorum*, *C. canis*, *C. muris*, *C. felis* and *C. suis* in immunocompetent individuals, particularly children [86, 95, 97, 103, 129, 141, 149, 153].

Subtyping studies of *Cryptosporidium* to date supports the dominance of anthroponotic transmission in African countries, despite close contact with farm animals. For example, a study conducted in children in the rural Ashanti region of Ghana reported that the human-to-human transmitted *C. hominis* subtype families Ia, Ib, Id and Ie made up 58.0% of all *Cryptosporidium* isolates typed, and within *C. parvum*, the largely anthroponotically transmitted subtypes families IIc and IIe, were detected in 42.0% of samples typed [98]. High levels of subtype diversity are also frequently reported, which is a common finding in developing countries and is thought to reflect intensive and stable anthroponotic *Cryptosporidium* transmission [6, 23, 89, 96, 98, 115, 141]. Similarly, another study in Kenyan children identified *C. hominis* subtypes in the majority of positives typed (82.8%), while the *C. parvum* IIc subtype family was identified in 18.8% of positives [115] (Table 2). To date, seven *C. hominis* subtype families (Ia, Ib, Id, Ie, If and Ih) have been identified in African countries (Table 2).

The mainly anthroponotically transmitted *C. parvum* IIc subtype family is the predominant subtype in sub-Saharan Africa, including Malawi, Nigeria, South Africa, and Uganda [87, 89, 98, 115, 141, 148, 152, 157]. However, it is important to note that the IIc subtype family has been detected in hedgehogs in Europe [158–160], suggesting potential zoonotic transmission.

In addition to the *C. parvum* IIc and the rarer anthroponotically transmitted IIe subtype family, a range of additional *C. parvum* subtype families (IIa, IIb, IId, IIg, Iii, IIh and IIm) have been identified in humans (Table 2). The *C. parvum* subtype family IIm, which was discovered in Nigeria [141], also appears to be anthroponotically transmitted, as it has not been identified in animals. High occurrences of zoonotic *C. parvum* subtype families (IIa and IId) have however been detected in some studies in Egypt, Ethiopia and Tunisia [23, 82, 95, 108, 140]. Few subtyping studies have been conducted on *C. meleagridis* isolates with *C. meleagridis* subtype IIIdA4 identified in humans in South Africa [89].

Recently a *gp60* subtyping assay has been developed for *C. viatorum* [161], the only species that to date has been found exclusively in humans. A single subtype family, XVa, was identified containing multiple alleles (XVaA3a-XVaA3f) [161]. A single case of XVaA3b originating in Kenya has been identified and nine samples from Ethiopia belonged to XVaA3d; however, this subtype is not a strictly African subtype as the same subtype was also identified in a United Kingdom patient with a history of traveling to Barbados [161]. Currently no animal reservoir has been identified for *C. viatorum*, but extensive studies of animals in the same areas where the human infections originated are required to clarify whether animal reservoirs exist.

The relative clinical impact of *C. hominis* and *C. parvum* in African communities is poorly defined. In a study in children under 15 years in Ghana, *C. hominis* infection was mainly associated with diarrhoea whereas *C. parvum* infection was associated with both diarrhoea and vomiting [98]. A study in Tanzania reported that *C. hominis* was the predominant species and was associated with a longer duration of symptoms, a higher rate of asymptomatic infection, and a lower CD4 cell count *versus C. parvum*-infected patients (*P* < 0.05) [162]. However, another study in Uganda reported that the vast majority of children presenting with diarrhoea lasting for 31 days or longer were HIV-positive and were infected with isolates belonging to the *C. parvum* subtype family Iii, followed by the *C. hominis* subtype Ie. The *C. parvum* IIc and IIg and *C. hominis* Ia, Ie, and Id subtype families were found in children with diarrhoea lasting for 21 days or less [148].

Relatively few *Giardia* genotyping studies have been conducted in Africa, however available reports reveal that five *G. duodenalis* assemblages (A, B, C, E and F) have been identified in humans (Table 3). In Africa, Assemblage B was the most prevalent among typed samples (19.5–100%) in 18 out of 28 studies reviewed (Table 3) with Assemblage A the dominant assemblage (1.4–100%) in the remaining 10 studies [96, 122, 163–167] (see Table 3). Although many studies have reported that *Giardia* is not associated with severe diarrhoea [8], one study reported that the prevalence of *G. duodenalis* Assemblage A was higher among children with vomiting and abdominal pain [22]. Assemblage C was detected in an adult immunocompromised male suffering from bladder cancer and diarrhoea in Egypt [121] and Assemblage F was reported in six diarrhoeal and one asymptomatic individual in Ethiopia [164]. In that study, four of the identified Assemblage F isolates were mixed infections with Assemblage A. Assemblage E has been reported in humans in three separate studies in Egypt with a prevalence of up to 62.5% in one study population [80, 168, 169]. Subtyping studies in Africa have identified subassemblages AI, AII, BIII, BIV and various novel subassemblages (Table 3).

## *Cryptosporidium* and *Giardia* in domesticated animals in Africa

In Africa, *Cryptosporidium* and *Giardia* have been reported in several domesticated animal species including cattle, sheep, goats, farmed buffalo, horses, poultry (chicken and turkey), pigs, cultured tilapia (fish) and dogs [26, 84, 93, 94, 97, 111, 123, 170–173]. However, the majority of research has been conducted on cattle. Prevalence ranging from < 1% in calves [154] to > 86% in calves [174] have been reported for *Cryptosporidium* and < 6% [123] to > 30% [175] prevalence for *Giardia* in adult cattle and calves, respectively. As with most studies, the prevalence of *Cryptosporidium* was greater in young animals (1 day to 3 months) than older ones. Age, source of drinking water and diarrhoea has been associated with *Cryptosporidium* prevalence in cattle [26, 118, 174]. For example, in a study in Egypt, calves watered with canal or underground water were at a higher risk of infection than calves watered with tap water [26].

*Cryptosporidium parvum, C. ryanae*, *C. bovis and C. andersoni* are the most common species detected in cattle (*Bos taurus* and *Bos indicus*), although *C. hominis*, *C. suis* and *Cryptosporidium* deer-like genotype have also been reported [26, 84, 90, 95, 97, 110, 112, 113, 118, 174, 176, 177]. Younger calves had a higher occurrence of *C. bovis* and *C. ryanae* while *C. parvum* seems to be dominant in pre-weaned calves [85, 112].

Although little research has been done in other domesticated animals, *C. ryanae*, *C. bovis* and *C. parvum* have been reported in farmed buffalos [95, 111, 113], *C. xiaoi*, *C. bovis* and *C. suis* in sheep [102, 113, 117, 178] and *C. xiaoi* and *C. parvum* in goats [102, 117] (Table 5). In addition, *C. parvum* and *C. suis* have been identified in pigs [97], *C. erinacei* in horses [94] and *C. canis* in dogs [97]. *Cryptosporidium meleagridis* was identified in both turkeys and chickens [93, 178] and *C. baileyi* has been identified in chickens [93]. All the species reported in domesticated animals, except for *C. ryanae* and *C. baileyi*, have been identified in humans from Africa [23, 25, 26, 97, 101, 102, 115, 139–142] (see Table 2), suggesting that domestic animals may act as zoonotic reservoirs for human infections. Humans working closely with farmed animals especially calves are known to be more at risk of zoonotic infection with *C. parvum* and may excrete oocysts without showing clinical symptoms and act as a source of infection for household members [118].

**Table 5:** Prevalence and *Cryptosporidium* species and subtypes reported from domesticated animals and wildlife in Africa

Country	Animal type	Animal species	Reported prevalence^a^ (No. positive/No. tested)	Species (Prevalence in %)^b^	*gp60* subtypes	Reference
Algeria	Domesticated	Turkey (*Meleagris gallopavo*)	44.6% (25/57)	*C. meleagridis* (100)	IIIgA18G4R1; IIIgA19G5R1; IIIgA20G4R1; IIIgA21G3R1^c^; IIIgA24G3R1; IIIgA26G2R1	[93]
		Chickens (*Gallus domesticus*)	34.4% (31/90)	*C. meleagridis* (83.9)	IIIgA18G4R1; IIIgA19G5R1^c^; IIIgA21G3R1; IIIgA24G2R1; IIIgA26G3R1^c^	
				*C. baileyi* (16.1)		
Algeria	Domesticated	Horse (*Equus caballus*)	2.9% (4/138)	*C. erinacei* (100)	XIIIaA22R9	[94]
Algeria	Domesticated	Horse (*Equus caballus*)	2.3% (5/219)	*C. parvum* (60.0)	IIaA16G1R1^c^	[100]
				*C. hominis* (20.0)	IkA15G1	
				*C. muris* RN66 (20.0)		
		Donkey (*Equus africanus asinus*)	1.6% (2/124)	*C. parvum* (50.0)	IIaA16G1R1	
				*C. muris* TS03 (50.0)		
Central Africa Republic	Wildlife	Wild western lowland gorillas (*Gorilla gorilla gorilla*)	0.5% (1/201)	*C. bovis* (100)		[122]
		African buffalo (*Syncerus caffer*)	5.0% (1/20)	*C. muris* TS03 (100)		
Côte d’Ivoire	Domestic	Chicken (*Gallus gallus*)	16.1% (5/31)	*C. meleagridis* (80.0)		[106]
				*C. parvum* (20.0)		
Egypt	Domesticated	Duck (*Anas platyrhynchos*)	39.9% (365/915)	*C. meleagridis* (100)		[353]
Egypt	Domesticated	Cattle (*Bos taurus*) and buffalo (*Bubalus bubalis*)	Cattle: 31.2% (185/593); Buffalo: 35.5% (75/211)	*C. parvum* (65.7)	IIaA15G1R1; IIdA19G1; IIdA20G1^c^	[26]
				*C. ryanae* (11.8)		
				*C. bovis* (4.1)		
				*C. parvum* + *C. ryanae* (11.2)		
				*C. parvum* + *C. bovis* (5.3)		
				*C. parvum* + *C. andersoni* (1.8%)		
Egypt	Domestic	Cattle (*Bos taurus*)	10.2% (49/480)	*C. parvum* (100)	IIdA20G1	[108]
		Buffalo (*Bubalus bubalis*)	12.3% (38/310)	*C. parvum* (100)	IIdA20G1	
Egypt	Domestic	Cattle (*Bos taurus*)	13.6% (269/1974)	*C. parvum* (43.5)	IIdA20G1; IIaA15G1R1^c^; IIaA14G1R1r1b	[112]
				*C. andersoni* (10.1)		
				*C. bovis* (10.1)		
				*C. ryanae* (18.8)		
				Mixed species (17.4)		
Egypt	Domestic	Water buffalo calves (*Bubalus bubalis*)	9.5% (17/179)	*C. parvum* (41.2)	IIdA20G^c^; IIaA15G1R1	[111]
				*C. ryanae* (58.8)		
Egypt	Domestic	Calves (*Bos taurus*)	30.2% (29/96)	*C. parvum* (92.3)	IIdA20G1^c^; IIaA15G2R1	[110]
				*C. andersoni* (7.7)		
Egypt		Buffalo (*Bubalus bubalis*)	1.3% (6/466)	*C. parvum* (33.3)	IIdA20G1; IIaA15G1R1	[113]
				*C. ryanae* (66.7)		
		Cattle (*Bos taurus*)	6.9% (31/450)	*C. parvum* (74.2)	IIdA20G1^c^; IIaA15G1R1	
				*C. ryanae* (16.1)		
				*C. bovis* (9.7)		
		Sheep (*Ovis aries*)	2.5% (3/120)	*C. xiaoi* (100)		
Egypt	Domestic	Cattle (*Bos taurus*)	10.2% (49/480)	*C. parvum* (100)	IIdA20G1	[108]
		Buffalo (*Bubalus bubalis*)	12.3% (38/310)	*C. parvum* (100)	IIdA20G1	
Ethiopia		Calves (*Bos taurus*)	15.8% (71/449)	*C. andersoni* (76.1)		[354]
				*C. bovis* (19.7)		
				*C. ryanae* (4.2)		
Kenya	Domestic	Calves and cattle (*Bos taurus*)	7.7% (134/1734)	*C. parvum-*like (68.0)		[355]
				*C. ryanae* (16.0)		
				*C. andersoni* (12.0)		
				*C. hominis* (4.0)		
Kenya	Domestic	Cattle (*Bos taurus*)	Not reported	*Cryptosporidium* deer-like genotype (100)		[177]
Kenya	Wildlife	Olive baboon (*Papio anubis*)	2.6% (6/235)	*C. hominis* (100)	IbA9G3^c^; IIfA12G2^c^; IiA14	[187]
Madagascar	Domestic	Cattle (*Bos taurus*)	29.0% (18/62)	*C. suis* (94.4); *Cryptosporidium* spp. (5.6)		[97]
		Pig (*Sus scrofa*)	23.5% (4/17)	*C. parvum* (75.0); *C. suis* (25.0%)		
		Rodents	33.3% (16/48)	Rat genotype III (31.0); Rat genotype IV (6.3); *C. meleagridis* (43.8); *C. suis* (6.3); Unknown (12.5)		
		Dog (*Canis lupus*)	100% (1/1)	*C. canis* (100)		
	Wildlife	Lemur	4.0% (1/25)	*C. hominis* (100)		
Nigeria	Domestic	Calves (*Bos taurus*)	52.3% (34/65)	*C. bovis* (52.9); *C. ryanae* (14.7); *C. bovis* and *C. ryanae* (32.4)		[356]
Nigeria	Domestic	Calves (*Bos taurus*)	16.0% (31/194)	*C. bovis* (45.2); *C. ryanae* (25.8); *C. andersoni* (16.1); *C. bovis* + *C. ryanae* (9.7); *C. bovis* + *C. andersoni* (3.2)		[85]
Rwanda	Wildlife	Mountain gorilla (*Gorilla beringei beringei*)	4.0% (4/100)	*C. muris* (50); *C. meleagridis* (50.0)		[182]
South Africa	Domestic	Calves (*Bos taurus*)	0.6% (2/352)	*C. parvum* (50.0); *C. bovis* (50.0)		[154]
South Africa	Domestic	Calves (*Bos taurus*)	8.0% (4/51)	*C. andersoni* (50.0); *C. bovis* (50.0)		[90]
	Wildlife	African buffalo (*Syncerus caffer*)	2.8% (2/71)	*C. ubiquitum* (50.0); *C. bovis* (50.0)		
		Impala (*Aepyceros melampus*)	2.8% (2/71)	*C. ubiquitum* (100)		
Tanzania	Wildlife	Baboons (*Papio* spp.)	10.6% (5/47)	*C. hominis* (100)	IfA12G2	[102]
		Chimpanzees (*Pan* spp.)	19.0% (16/84)	*C. hominis* (68.8); *C. suis* (25.0); *C. suis* and *C. hominis* (6.2)	IfA12G2	
	Domestic	Sheep (*Ovis aries*)	22.2% (2/9)	*C. xiaoi* (100)		
		Goats (*Capra aegagrus hircus*)	13.9% (5/56)	*C. xiaoi* (100)		
Tunisia	Domestic	Calves (*Bos taurus*)	21.4% (15/70)	*C. parvum* (100)	IIaA15G2R1^c^; IIaA16G2R1; IIaA13G2R1; IIaA20G3R1; IIdA16G1	[114]
Tunisia	Domestic	Sheep (*Ovis aries*)	11.2% (10/89)	*C. bovis* (100)		[178]
		Chicken (*Gallus gallus domesticus*)	4.5% (9/200)	*C. meleagridis* (100)		
Tunisia	Domestic	Calves (*Bos taurus*)	86.7% (26/30)	*C. parvum* (100)		[174]
Uganda	Wildlife	Black-and-white colobus (*Colobus guereza*;)	3.4% (1/29)	*C. parvum* (100)		[107]
		Red colobus (*Procolobus rufomitratus*)	26.7% (8/30)	*C. parvum* (75.0%); *C. parvum*/*C. hominis/C. cuniculus* (25.0)		
	Domestic	Goats (*Capra aegagrus hircus*)	3.5% (2/57)	*C. parvum* (100)		
Uganda	Wildlife	Free-ranging mountain gorilla (*Gorilla gorilla beringei*)	4.0% (4/100)	*C. parvum* (100)		[104]
Zambia	Domestic	Calves (*Bos taurus*)	34.0% (70/207)	*C. parvum* (61.9)		[118]
				*C. bovis* (33.3)		
				*Cryptosporidium* deer-like genotype (4.8)		
Zambia		Goat kid (*Capra aegagrushircus*)	4.8% (5/105)	*C. parvum* (100)		[117]
		Lamb (*Ovis aries*)	12.5% (19/152)	*C. parvum* (83.3)		
				*C. suis* (16.7)		
Zambia		Calves (*Bos taurus*)	19.2% (142/744)	*C. parvum* (64.4)		[116]
				*C. bovis* (33.3)		
				*C. suis* (2.2)		

^a^Prevalence (percentage of number positive out of the total number of animal species tested)

^b^Prevalence (percentage of species identified out of the total number of species genotyped per animal)

^c^Dominant *gp60* subtype

Subtyping of *C. parvum* from animals at the *gp60* locus identified *C. parvum* subtypes IIa and IId, with IIaA15G1R1, IIaA15G2R1 and IIdA20G1 the most common [95, 110–113] (see Table 5). A unique subtype IIaA14G1R1r1b was also isolated from a calf in Egypt [112]. *Cryptosporidium erinacei* subtype XIIIa was found in horses from Algeria [94]. In a study in rural Madagascar, peri-domestic rodents were found to be infected with *Cryptosporidium* rat genotype III, rat genotype IV, *C. meleagridis*, *C. suis* and 2 unknown genotypes [97].

*Giardia duodenalis* Assemblage E is the dominant species in ruminant livestock (cattle, farmed buffalo and goats) from the Central African Republic, Egypt, Rwanda, Tanzania and Uganda [80, 120, 122–124, 169, 175, 179]. Assemblage A (subtypes AI and AII) has been reported in goats, cattle, buffalos, ducks and chickens from Cote d’Ivoire, Egypt, Tanzania and Uganda and Assemblage B (BIV) and/or Assemblage A and B have been reported in goats, ducks and cattle from Cote d’Ivoire, and Tanzania [80, 106, 120, 123, 169, 175, 179]. Assemblage A was also identified in cultured tilapia and mullet (*Tilapia nilotica* and *Mugil cephalus*, respectively) from Egypt [171].

## *Cryptosporidium* and *Giardia* in African wildlife

The majority of studies on *Cryptosporidium* and *Giardia* in African wildlife have been conducted in wildlife parks. These studies have included western lowland gorillas from the Lope National Park in Gabon [180], mountain gorillas from the Bwindi Impenetrable National Park in Uganda and the Volcanoes National Park in Rwanda [104, 124, 163, 181, 182], chimpanzees from Tanzania, elephants, buffalos and impalas from the Kruger National Park, South Africa [90, 183], olive baboons from the Bwindi Impenetrable National Park, Uganda [184] and bamboo lemurs and eastern rufous mouse lemurs from the Ranomafana National Park, Madagascar [97, 185]. In addition, *Cryptosporidium* oocysts and *Giardia* (oo)cysts together with other gastrointestinal parasites (*Nasitrema attenuata*, *Zalophotrema* spp. and *Pholeter gasterophilus*) were found in dolphins in Egypt, but no genotyping was conducted [186].

*Cryptosporidium hominis* was reported in olive baboons from Kenya and Tanzania and in lemurs from Madagascar, suggesting possible spill-back from humans. *Cryptosporidium hominis* and *C. suis* has been reported in chimpanzees from Tanzania and [97, 102, 187] and *C. parvum* was reported in gorillas from Uganda [104]. Subtyping at the *gp60* locus identified *C. hominis* subtypes IfA12G2 (the commonest), IbA9G3 and a novel subtype IiA14 in olive baboons and chimpanzees from Kenya and Tanzania, respectively [102, 187]. In wild ruminants, *C. ubiquitum* and *C. bovis* has been identified in forest buffalos and *C. ubiquitum* in Impala from South Africa [90]. *Cryptosporidium ubiquitum* is considered an emerging zoonotic pathogen [188] and has been reported in humans in Africa in Nigeria [86, 141] and increasing human encroachment into wildlife-populated areas in Africa, is likely to increase zoonotic transmission.

*Giardia duodenalis* Assemblage A and B (subtypes BIII and BIV) have been reported in gorillas from Uganda and Rwanda, respectively [124, 163] and Assemblage B in usrine colobus monkey from Ghana [189] (Table 4), again suggesting spill-back. *Giardia duodenalis* cysts have been found in the faeces of other animals including grasscutters (*Thryonomys swinderianus)* [190], but no genotyping was done. Almost all the *Cryptosporidium* and *Giardia* species identified in wildlife are infectious to humans with potential for zoonosis or spill-back from humans to animals. For example, a high prevalence of cryptosporidiosis was reported in park staff members (21%) who had frequent contact with gorillas *versus* 3% disease prevalence in the local community in Uganda [191].

## Waterborne and foodborne cryptosporidiosis and giardiasis in Africa

As *Cryptosporidium* and *Giardia* (oo)cysts are robust and resistant to environmental conditions, including disinfectants such as chlorine used in water treatment systems, numerous waterborne and foodborne outbreaks of human cryptosporidiosis and giardiasis have been reported, with *Cryptosporidium* and *Giardia* responsible for > 95% of outbreaks worldwide [192–202].

Relatively little is known about the presence and prevalence of *Cryptosporidium* and *Giardia* in food and water in Africa. Both parasites have been detected in food such as fresh fruits and vegetables in Ethiopia, Egypt, Ghana, Libya and Sudan [203–207], and Tiger nuts (*Cyperus esculentus*) from Ghana [208]. *Cryptosporidium* was detected in 16.8% of reared black mussels (*Mytilus galloprovincialis*) in Mali [209]. *Cryptosporidium* does not multiply in bivalves, but they can be an effective transmission vehicle for *Cryptosporidium* oocysts, especially within 24–72 h of contamination, with viable oocysts present in bivalves up to 7 days post infection [210]. *Cryptosporidium* and *Giardia* (oo)cysts were identified from 34.3% and 2.0% of coins and 28.2% and 1.9% of bank notes (respectively) used by food-related workers in Alexandria, Egypt [211]. As coins and banknotes are some of the objects most handled and exchanged by people, this raises the potential of parasite transmission even between countries.

In many rural African households, untreated water is used for various purposes such as bathing, cooking, drinking and swimming, often exposing them to waterborne *Cryptosporidium* and *Giardia* [212, 213]. More than 300 million people in sub-Saharan Africa have poor access to safe water, predisposing them to infections from waterborne pathogens, and cryptosporidial infections are known to be prevalent among communities which lack access to clean potable water supply [214–216]. Poverty is therefore a key limiting factor to accessing safe water. In many communities, particularly those in rural areas where the average income is ~ US$1 per person per day [217], individuals have limited access to privately owned water resources that provide safe water [218]. This, coupled with inadequate water treatment, poor hygiene practices, drinking unboiled water and lack of education programmes, predisposes many rural African communities to cryptosporidiosis and giardiasis [218].

*Cryptosporidium* oocysts and *Giardia* cysts have been detected in a variety of African water sources including irrigation water in Burkina Faso [219], a stream, well, spring and lake in Cameroon [220, 221], wastewater in Côte d’Ivoire [222], packaged drinking water in Ghana [223–225], tap water, drinking water treatment plants, canals, tanks and swimming pools in Egypt [226–230]. They have also been detected in water sources (surface and well), treated water storage tanks and tap water in Ethiopia [231, 232], the Kathita and Kiina rivers and surface water in Kenya [233, 234], water from wells and the Kano river in Nigeria [235], the surface waters of the Vaal Dam system [236], treated and untreated effluents, sewage, drinking water and roof-harvested rainwater in South Africa [237–240]. In Tunisia, they have been detected in watersheds, treated, raw wastewater and sludge samples [241, 242], in Uganda, in natural and communal piped tap water from the Queen Elizabeth protected area [218], in piped water in Zambia [243, 244] and wells, springs, tap water and rivers in Zimbabwe [245].

Genotyping of *Cryptosporidium* and *Giardia* from these water sources identified *C. parvum* from the Kathita and Kiina rivers, *C. parvum* and *C. andersoni* in Muru regional surface waters (both in Kenya) [233], *C. hominis* and *Giardia* Assemblages A and B in sewage treatment plants from South Africa [238, 240]. *C. hominis* (subtypes IdA15G1, IaA27R3), *C. parvum* (subtypes IIaA21, IIcA5G3b), *C. muris*, *C. andersoni*, *Giardia* Assemblage A (subtypes A1 and AII), B and a novel *Giardia* subtype were isolated from treated, raw wastewater and sludge samples in Tunisia [241]. In addition, *C. parvum* (subtypes IIaA15G2R1, IIaA17G2R1, IIaA18G3R1, IIaA20G2R1, IIaA21R1, IIaA21G2R1, IIcA5G3b), *C. muris*, *C. andersoni*, *C. hominis* (subtypes IaA26R3, IaA27R4, IdA14), *C. ubiquitum*, *Cryptosporidium* rat genotype IV, novel *Cryptosporidium* genotypes, *C. meleagridis*, avian genotype II, *Giardia* Assemblage A (subtypes AI and AII), Assemblages B and E were isolated from treated and raw wastewater plants and sludge samples, also from Tunisia [242]. In the latter report, the most prevalent genotypes were Assemblage A (86.8%) and *C. andersoni* (41.2%) out of 99 *Giardia* and 114 *Cryptosporidium*-positive PCR products, respectively.

## Treatment of cryptosporidiosis and giardiasis in Africa

Another contributing factor to the high prevalence and widespread distribution of *Cryptosporidium* and *Giardia* in Africa is the lack of treatment options. Currently no effective vaccine exists for *Cryptosporidium* and only one drug, nitazoxanide (NTZ, Alinia; Romark Laboratories, Tampa, Florida, USA), is available for use against *Cryptosporidium*. This drug, however, is currently not recommended for use in infants < 12 months of age, exhibits only moderate clinical efficacy in malnourished children and immunocompetent people, and none in immunocompromised individuals like people with HIV [246, 247]. In 2015, > 25 million adults and children were infected with HIV/AIDS in Africa [2], and the UN Food and Agriculture Organization estimates that 233 million people in sub-Saharan Africa were malnourished in 2014–6 [248]. The ineffectiveness of nitazoxanide in HIV-positive individuals and the contribution of malnourishment to impaired immunity [30], means that nitazoxanide is ineffective against the most important target population in Africa. In individuals co-infected with HIV, antiretroviral therapy (ART) has been successful in controlling chronic diarrhoea and wasting due to cryptosporidiosis [27, 249, 250]. Currently, supportive care and ART (for HIV/AIDS patients) form the basis for treatment of cryptosporidiosis.

As with *Cryptosporidium*, a human vaccine for giardiasis is not available. Several classes of antimicrobial drugs are available for the treatment of giardiasis. The most commonly utilised worldwide are members of the 5-nitroimidazole (5-NI) family such as metronidazole and tinidazole. However, this first line therapy fails in up to 20% of cases and cross-resistance between different agents can occur [251], and resistance to all major antigiardial drugs has been reported [252]. Albendazole is also effective in treating giardiasis [251, 253], although its efficacy varies markedly (25–90%), depending on the dosing regimen [254]. Nitazoxanide has been shown to reduce symptom duration in individuals with giardiasis [255] and quinacrine, an old malaria drug, reportedly has 90% efficacy against giardiasis [256], but has potentially severe adverse effects, including a number of psychiatric and dermatologic manifestations [254]. For *Cryptosporidium*, new classes of more effective drugs are a high priority and for *Giardia*, improvements in potency and dosing of currently available drugs, and the ability to overcome existing and prevent new forms of drug resistance, are priorities in antigiardial drug development [254].

The prohibitive cost of *de novo* drug development, estimated to be between $500 million and $2 billion per compound successfully brought to market [257], is another major limiting factor in the development of anti-cryptosporidial and anti-giardial drugs. Treatment of *Cryptosporidium* and *Giardia* in African countries, despite having a large target population, has a small market in the developed world and pharmaceutical companies are often hesitant to invest in costly *de novo* campaigns to develop new therapeutics for developing countries. Therefore, the primary challenge for further drug development is the underlying economics, as both parasitic infections are considered Neglected Diseases with low funding priority and limited commercial interest [254]. For this reason, there has been a movement to ‘repurpose’ existing therapeutics for off-label applications, as repurposed drugs cost around 60% less to bring to market than drugs developed *de novo* [258]. For example, drugs such as the human 3-hydroxy-3-methyl-glutaryl-coenzyme A (HMG-CoA) reductase inhibitor, itavastatin and auranofin (Ridaura®) were initially approved for the treatment of rheumatoid arthritis and have been shown to be effective against *Cryptosporidium* in vitro [259, 260], which holds promise for future anticryptosporidial drugs.

## The impact of climate change and HIV status on cryptosporidiosis and giardiasis in Africa

Waterborne transmission is a major mode of transmission for both *Cryptosporidium* and *Giardia*. Climate change represents a major threat for access to safe drinking water in Africa which has more climate sensitive economies than any other continent [261]. Increasingly variable rainfall patterns are likely to affect the supply of fresh water in Africa. Some regions in Africa have become drier during the last century (e.g. the Sahel) [262] and by the 2090s, climate change is likely to widen the area affected by drought, double the frequency of extreme droughts and increase their average duration six-fold [263]. Climate change will also increase levels of malnutrition in Africa, as it will lead to changes in crop yield, higher food prices and therefore lower affordability of food, reduced calorie availability, and growing childhood malnutrition in Sub-Saharan Africa [264]. Malnutrition in turn undermines the resilience of vulnerable populations to cryptosporidial and giardial infections, decreasing their ability to cope and adapt to the consequences of climate change.

Surface water concentrations of *Cryptosporidium* and *Giardia* in Africa are also expected to increase with increased population growth. The Global Waterborne Pathogen model for human *Cryptosporidium* emissions, predicts that while *Cryptosporidium* emissions in developing countries will decrease by 24% in 2050, in Africa, emissions to surface water will increase by up to 70% [265]. Given the lack of treatment options, particularly for *Cryptosporidium*, high-level community awareness, policy formulations and regular surveillance are needed in order to limit the waterborne, zoonotic and anthroponotic transmission of *Cryptosporidium* and *Giardia*.

This cannot be achieved, however, unless there is a commitment from African governments to supply clean potable water, particularly to rural communities, improve sanitation by connecting the population to sewers and improve waste water treatment. Community programmes must be initiated to educate the people on water safety measures, personal hygiene and water treatment processes. The achievement of these goals hinges on the elimination of malnutrition and a significant reduction in HIV levels in African populations. The introduction of ART in HIV patients which partially restores the immune function has been important in reducing the prevalence of *Cryptosporidium* in HIV patients [266, 267]. Furthermore, it has been suggested that HIV protease inhibitors can act as antiparasitic drugs. For example, in experimental studies, the drugs indinavir, saquinavir, and ritonavir have been reported to have anti-*Cryptosporidium* spp. effects both in vitro and in vivo [268]. However, most African government have not invested sufficient funds and resources to ensuring alleviation of malnutrition and HIV [261, 269] and many HIV-prevention services still do not reach most of those in need [269], largely due to under-staffing of, and the poor geographical distribution of available services for those in need.

Despite the millennium development goals target to reduce hunger by half by 2015, major failures have been recorded in Africa. Out of the > 800 million people still suffering from hunger in the world, over 204 million come from Sub-Saharan Africa. The situation is currently getting worse in this region as it moved from 170.4 million hungry people in 1990 to 204 million in 2002 [270]. This increase has generally been attributed to poverty, illiteracy, ignorance, big family sizes, climate change, policy and corruption [261].

## Conclusions

*Cryptosporidium* and *Giardia* are prevalent in both humans and animals in Africa with both anthroponotic and zoonotic transmission cycles. *Cryptosporidium* is unequivocally associated with moderate-to-severe diarrhoea in African children but further studies are required to determine if *Giardia* infections in early infancy are positively linked to moderate-to-severe diarrhoea, whether some paediatric hosts (e.g. more stunted) are more prone to develop persistent diarrhoea, whether *Giardia* decreases the risk of acute diarrhoea from other specific enteropathogens, and whether specific *Giardia* assemblages exhibit enhanced pathogenicity over other assemblages and subassemblages. Efforts in reducing HIV in African countries should focus on earlier identification of HIV, providing earlier access to ART and improved case management for HIV-infected individuals (particularly children) and reducing the cultural and social stigma directed at persons living with HIV/AIDS. “One Health” initiatives involving multidisciplinary teams of veterinarians, medical workers, relevant government authorities, water and sanitation engineers, water managers and public health specialists working together are essential for the control and prevention of cryptosporidiosis and giardiasis in African countries.

## Abbreviations

AIDS: Acquired immunodeficiency virus syndrome
ART: Antiretroviral therapy
*bg*: Beta-giardin
COWP: *Cryptosporidium* oocyst wall protein
*ef1-α*: Elongation factor 1-alpha
ELISA: Enzyme linked immunosorbent assay
*gdh*: Glutamate dehydrogenase
GEMS: Global enteric multicenter study
*gp60*: Glycoprotein 60
HIV: Human immunodeficiency virus
HMG-CoA: Human 3-hydroxy-3-methyl-glutaryl-coenzyme A
HSP70: Heat shock protein 70
ITS: Internal transcribed spacer
LAMP: Loop-mediated isothermal amplification
qPCR: Quantitative real-time polymerase chain reaction
rDNA: Ribosomal deoxyribonucleic acid
RFLP: Restriction fragment length polymorphism
rRNA: Ribosomal ribonucleic acid
*tpi*: Triose phosphate isomerase
TRAP: Thrombospondin-related adhesive protein
